# Does psychological strengths and subjective well-being predicting parental involvement and problem solving among Malaysian and Indian students?

**DOI:** 10.1186/2193-1801-3-756

**Published:** 2014-12-19

**Authors:** Aqeel Khan, Roslee Ahmad, Abdul Rahim Hamdan, Mohamed Sharif Mustaffa, Lokman Mohd Tahir

**Affiliations:** Faculty of Education, Universiti Teknologi Malaysia (UTM), Skudai, 81310 Johor Malaysia

**Keywords:** Psychological strengths, Well-being, Problem solving, Students

## Abstract

The present study examined the predictors of psychological strengths and subjective well-being for dealing with academic stress perceived by university engineering students. Sample of 400 Malaysian (N = 180 boys and N = 220 girls) age varies 18 to 25 years and 400 Indian students (N = 240 boys and N = 160 girls) age varies 18 to 25 years from public universities were participated. Quantitative method was used for data analysis. Findings shows that gender, religiosity and socioeconomic status are significantly influencing psychological strengths and subjective well-being of both Indian and Malaysian students. Findings also revealed that parental involvement and problem solving coping styles were significantly predicting psychological strengths and subjective well-being among both countries participants. Findings of the current study provide the insight for the educators, and parents dealing with adolescents.

## Introduction

Malaysian culture represents diverse multi-ethnic and multi-cultural society (Verkuyten & Khan^2012^; cited in Khan et al.2014a; Janssens et al.^2014^ & Ghani et al.^2014^) and India as well represents multi religious society (Husain et al.^2012^). Stresses faced by among Malaysian and Indian students, like academic stress emerges as significant mental health problem in recent years (Chou et al.^2011^ & Rangaswamy^1995^; Khan et al.2014b). In Asian context, parents hope their children will get good grades because it is important in getting a good job. For example, there is an old proverb in Chinese “Everything is unworthy except studying” (cited in Khan^2012^). Therefore emerging adolescents grow up believing that academic failure will have negative repercussions, so they are putting immense pressure on themselves to excel in university they were also cognizant to live up to the expectations of parents and teachers (Ang & Huan^2006^). Parental involvement greatly influences subjective well-being of the adolescents. Existing researches showed strong relationship of parental involvement and subjective well-being (Dmitrieva et al.^2004^; Doyle & Markiewicz^2005^; Gibson & Jefferson^2006^; Wilkinson^2004^).

According to Diener et al. (2006), subjective well-being (SWB) may regard as one’s evaluations of their own lives, which can be their judgments towards current status quo like life satisfaction and evaluations based on personal feelings (including because of the way they evaluate their lives either as going in a good way or imperfectly. Research on SWB consisted of a worldwide life satisfaction which including positive affect and negative affect (Lightsey^1996^; Robbins & Kliewer^2000^). Even though there were many researches had been carried out on the topic of SWB in recent years, there still an overall lack of studies that have examined the experience of SWB in children and adolescents (Lent et al.^2005^) moods and inner emotions).

Various studies report that Asians give more priority to academic achievement since it brings career success (Alshemmeri et al.^2011^; Gloria & Ho^2003^; Sue & Okazaki^1990^). Utilizing effective coping strategies, parental involvement and positive psychological strengths can help alleviate the negative effects of stress (Dressler^1991^) and increase the well-being, while deficits in problem-solving behaviors will increase the risk of academic stress. According to Noack and Puscher (1999), high parental support has been associated with positive guidance for emerging adolescent (Khan & Kalu^2011^). Parent support also allow the emerging adolescent to cope better with the transition to adolescent (Scabini et al.^1999^).

Present study examines (1) Influence of demographic characteristics on the outcome variables (2) relationship of positive psychological strengths and its dimensions with subjective well-being, parental involvement, problem solving coping and academic stress; and (3) the role of parental involvement and problem solving coping as a predictor of positive psychological strengths and subjective well-being were investigated.

## Method

### Participants

Firstly informed consent were obtained from the participants and ethical approval granted by university. Survey data were collected from 400 Malaysian engineering students comprising 220 girls and 180 boys between the ages of 18–25 years (Mage =20.01 years, SD =13.97), residing in Kuala Lumpur and Johor Bahru cities of Malaysia. Another group of participants includes 400 Indian students, 160 girls and 240 boys between the ages of 18–25 (Mage =22.01 years, SD =15.22), residing in New Delhi city of India.

### Measures

The following measures were used:

*Positive psychological strengths questionnaire (PPSQ)*; (Luthans et al.2007a). The 24 items of positive psychological strengths with six items of each sub-measure includes self efficacy, hope, optimism and resiliency. The scale items were anchored from “1” (strongly disagree) to “6” (strongly agree). Example of items include “I feel confident helping to set targets/goals in my work area” (confidence); “I can think of many ways to reach my current work goals” (hope); “When things are uncertain for me at work I usually expect the best” (optimism), and “I usually take stressful things at work in stride” (resiliency). Luthans et al.2007b; alpha = .88), used to measure the positive psychological strengths (Hope, Optimism, Resiliency and Self- efficacy) with response choices into a 6-point Likert-type scale (1 = strongly disagree, 2 = disagree, 3 = somewhat disagree, 4 = somewhat agree, 5 = agree, 6 = strongly agree). Reliability coefficients of current study for all the components were reported (Malaysian α = .84; Indian α = 87).

*Satisfaction with life scale (SWLS)*; (Diener et al.^1985^) consisting 5 items was used to measure the subjective well being. Subjects responded to items using a 7-point Likert scale ranging from 1 = “strongly disagree” to 7 = “strongly agree.” Responses were summed to produce a total SWLS score, with higher scores indicating more life satisfaction. Internal consistency (.87), test-retest reliability (.82, eight weeks), and validity of the SWLS are good (Diener et al.^1985^). The Cronbach’s alpha in the present study were observed (Malaysian α = .93; Indian α = 85).

*Student Academic Stress Scale* (Gadzella^1991^); The SASS used with four domains: Physiological, Behavioral, Cognitive, and Affective. The Student-life Stress Inventory (SSI) has been used in numerous studies to measure college student stress and to determine the reliability and validity of the instrument (Gadzella^1991^,1994a,1994b,^2004^; Gadzella & Baloglu^2001^). Cronbach’s alpha ranging from .52 to .85. The Cronbach’s alpha in the present study were observed (Malaysian α = .88; Indian α = 81).

*Parental Home Involvement* (Nyarko^2008^); Measures parental actions such as encouragement of their children to succeed, monitoring of their homework, going on outings with the participants. The scale was measured on a five-point likert scale ranging from 1-almost never to 5-very often. Some of the items on the scale are “My parents discuss my school progress with me”, “My parents go on outings with me.” In all, seven items were measured on this scale. The alpha coefficients were: Mother = 0.82 and father = 0.80. The Cronbach’s alpha in the present study were observed (Malaysian α = .90; Indian α = .94).

*Adolescent Coping Scale (ACS)* (Frydenberg & Lewis^1993^); It consists 6 items *Solving the Problem* subscale from the short version of ACS. High scores on the Solving the Problem indicate positive coping strategies. Internal consistency of the 18 scales has been reported to have alphas ranging from .45 to .85. Test retest reliability over a two week period has been shown to be moderate (Frydenberg and Lewis^1993^). The Cronbach’s alpha in the present study were observed (Malaysian α = .83; Indian α = 89).

*Demographic variables*; Participants gender, marital status, believe in religion or not and socio-economic status were also identified.

### Research question 1: What are the influence of gender, socio-economic status, and religiosity on positive psychological strengths and subjective well-being among Malaysian and Indian participants?

Table 1 represents the Influence of demographic characteristics of participants on outcome variables. The F-tests showed significantly higher level of positive psychological strengths among Indian male (Mean =14.39, SD =5.98) than Female (Mean =7.11, SD =3.44) (F =19.84, p < .001). There were significantly higher level of positive psychological strengths among participants of non- religious group (Mean =15.05, SD =2.96) than religious group (Mean =9.78, SD =3.25), (F =21.02, p < .001). There were significantly higher level of positive psychological strengths among participants belong to wealthier families (Mean =18.05, SD =6.20) than belong to poor families (Mean =6.45, SD =2.05), (F =7.97. p < .05).

**Table 1:** **Influence of demographic characteristics of participants on positive psychological strengths**

Positive psychological strengths (Malaysian)					Positive psychological strengths (Indian)			
	N	Mean	SD	F	N	Mean	SD	F
**Gender**								
Male	160	7.55	2.10	6.90*	220	14.39	5.98	19.84**
Female	240	4.02	0.84		180	07.11	3.44	
**Religiosity**								
Religious	286	8.17	2.96	17.14**	270	15.05	2.96	21.02**
Non-Religious	114	5.08	1.18		130	9.78	3.25	
**Socio-economic status**								
Belong to Wealthier Family	230	8.98	2.45	19.25**	267	18.05	6.20	07.97*
Belong to Poor Family	170	5.25	1.98		133	06.45	2.05	

*sig at .05; **sig at .001 level.

Among Malaysian male participants’ positive psychological strengths are higher level (Mean =7.55, SD =2.10) than Female (Mean =4.02, SD = .84) (F =6.95, p < .05). There are significantly higher level of positive psychological strengths among participants of religious group (Mean =8.17, SD =2.96) than non-religious group (Mean =5.08, SD =1.18), (F =17.14, p < .001). There are significantly higher levels of positive psychological strengths among participants belong to wealthier families (Mean =8.98, SD =5.25) than participants belong to poor families (Mean =5.25, SD =1.98), (F =19.25, p < .001).

Table 2 represents the Influence of demographic characteristics of participants on subjective well-being. The F-tests shows significantly higher level of subjective well-being among Indian male (Mean =14.39, SD =5.98) than Female (Mean =7.11, SD =3.44), (F =19.84, p < .001). There are significantly higher levels of subjective well-being among participants of religious group (Mean =15.05, SD =2.96) than non-religious group (Mean =9.78, SD =3.25), (F =21.02, p < .001). There are significantly higher level of subjective well-being among participants belong to wealthier families (Mean =18.05, SD =6.20) than poor families (Mean =6.45, SD =2.05), (F =7.97, p < .01).

**Table 2:** **Influence of demographic characteristics of participants on subjective well**-**being**

Subjective well-being (Malaysian)					Subjective well-being (Indian)			
	N	Mean	SD	F	N	Mean	SD	F
**Gender**								
Male	160	7.55	2.10	6.95*	220	14.39	5.98	19.84**
Female	240	4.02	0.84		180	07.11	3.44	
**Religiosity**								
Religious	286	8.17	2.96	17.14**	270	15.05	2.96	21.02**
Non-Religious	114	5.08	1.18		130	9.78	3.25	
**Socio-economic status**								
Belong to Wealthier Family	230	8.98	2.45	19.25**	267	18.05	6.20	07.97**
Belong to Poor Family	170	5.25	1.98		133	06.45	2.05	

*sig at .05; **sig at .001 level.

Among Malaysian male participants’ subjective well-being are higher (Mean =7.55, SD =2.10) than female (Mean =4.02, SD = .84), (F =6.95, p < .01). There are significantly higher levels of subjective well-being among participants of religious group (Mean =8.17, SD =2.96) than non-religious group (Mean =5.08, SD =1.18), (F =17.14, p < .001). There are significantly higher level of subjective well-being among participants belong to wealthier families (Mean = 8.98, SD = 5.25) than poor families (Mean = 5.25, SD = 1.98), (F = 19.25, p < .001).

### Research question 2: what are the relationship of independent and outcome variables among Malaysian and Indian participants?

Table 3 presents mean, Cronbach’s alpha reliability and intercorrelation matrix of the studied variables. Cronbach’s alpha reports that all variables are reliable from range .81 to .94. Most of the variables are positively correlated with each other except academic stress is negatively correlated with positive psychological strengths, subjective well-being, parental involvement, and problem solving. Based on the results of Malaysians, parental involvement had the positive correlation with problem solving coping with the coefficient of .41. On the other hand, for Indians participants, parental involvement also had the positive correlation with problem solving coping with the coefficient .32.

**Table 3:** **Means and intercorrelations of independent and outcome variables**

Variable	M	Α	1	2	3	4	5
Malaysians (n =400)*							
1. Positive psychological strengths	11.08	.84	1				
2. Subjective well-being	12.57	.93	.23**	1			
3. Parental involvement	16.28	.90	.27**	.34*	1		
4. Problem solving	14.33	.83	.33**	.21*	.41*	1	
5. Academic stress	6.87	.88	-.43	-.21	-.31	-.13	1
Indians (n =400)*							
1. Positive psychological strengths	12.99	.87	1				
2. Subjective well-being	18.42	.85	.44**	1			
3. Parental involvement	14.54	.94	.22*	.23*	1		
4. Problem solving	15.36	.89	.24**	.29**	.32**	1	
5. Academic stress	09.41	.81	-.06	-.12	-.10	.14	1

*sig at .05; **sig at .001 level.

### Research question 3: What are the role of parental involvement and problem solving coping as a predictor of positive psychological strengths and subjective well-being?

Tables 4 and5 reports regression model to test the hypothesis that gender, religiosity, socioeconomic status, parental involvement and problem solving significantly predicts positive psychological strengths and subjective well-being.

**Table 4:** **Regression model predicting positive psychological strengths and subjective well**-**being for Malaysian participants**

	Model 1			Model 2		
Predictors	ß	Std. Error	R^2^	ß	Std. Error	R^2^
Gender	.294*	.113		.263*	.193	
Religiosity	.372**	.106		.248*	.112	
Socioeconomic						
Status	.402**	.110		.439**	.131	
Parental Involvement	.422**	.163	.35	.632**	.147	.39
Problem Solving	.387**	.177		.432**	.116	

* p\0.05;** p\0.01.

Model 1 Dependent Variable: Positive Psychological Strengths.

Model 2 Dependent Variable: Subjective Well-Being.

**Table 5:** **Regression model predicting positive psychological strengths and subjective well**-**being for Indian participants**

	Model 1			Model 2		
Predictors	ß	Std. error	R^2^	ß	Std. error	R^2^
Gender	.284*	.125		.347*	.112	
Religiosity	.363**	.118		.233*	.132	
Socioeconomic						
Status	.443**	.166				
Parental involvement	.433**	.164	.27	.665**	.122	.38
Problem solving	.298**	.131		.254**	.123	

*p\0.05; **p\0.01.

Model 1 Dependent Variable: Positive Psychological Strengths.

Model 2 Dependent Variable: Subjective Well-Being.

Table 4 shows regression results that there is 35% of the variation exists in positive psychological strengths and 39% of the variation in subjective well-being can be explained including problem solving, parental involvement and demographic variables for Malaysian participants.

Table 5 shows 27% of the variation in positive psychological strengths and 38% of the variation in subjective well-being explained by problem solving, parental involvement and demographic variables for Indian participants.

## Discussion

Research findings declared higher positive psychological strengths and subjective well-being found for those who were: male, non-religious, and wealthy families of Indian subcontinent participants. Based on the analyzed results, Indian male were experiencing higher level of positive psychological strengths than Indian female. Previous studies revealed Indian female reported restriction in exposed to outside environment and they are also restricted to direct their feelings (Singh & Udainiya^2009^). Which clearly indicates that Indian women still discouraged and reflecting deprived part of the society. This explains why Indian women not holding positive attitudes towards oneself than their male counterpart (As cited Khan^2013^, p1290). Malaysian and Indian religious participants are scored significantly higher levels of positive psychological strengths and subjective well-being than non-religious group. Finding of Indian and Malaysian families conveyed significantly higher level of positive psychological strengths among participants who belong to wealthier families than belong to poor families (Diener et al.^1999^).

Findings show higher positive psychological strengths and subjective well-being for those who were: women, religious, and wealthy families of Malaysian subcontinent participants. Based on these findings which clearly showing that Malaysian Muslim culture rapidly changing the societal view and females are not feeling restricted and they are holding positive attitudes towards oneself, getting involved in friendship, having autonomous thought and actions, have the ability to manage complex environments to suit personal needs and values, recognizing life purpose, and continue developing their strengths. However, since Malaysia’s independence in 1957, women’s entry into the paid labor force has been dramatic, from 30.8% in 1957 to 47.1% in 1995 (Seventh Malaysia Plan, 1996–2000). The main reasons can be explained as (a) greater educational opportunities for women; (b) the implementation of the New Economic Policy by the government in 1969; and (c) the rapid economic development and industrialization, which have created jobs for women within the labor-intensive industries, such as electronics and textiles. Therefore education provides an opportunity among Malaysian women in breaking their barrier of success. Women are, in this broadened perspective, not passive recipients of welfare-enhancing help brought about by society, but are active promoters and facilitators of social transformations. Such transformations influence, of course, the lives and well-being of Malaysian women.

Religiosity also considered important determinant for deriving well-being (Stavrova et al.^2013^; Ellison et al.^2009^; Kim^2003^; Ventis^1995^). Religious Malaysian women reported higher level of positive psychological strengths than non-religious group (Khan^2013^). Frey and Stutzer (2002) reported strong link of religion and life satisfaction because of the mosque attendance provide opportunity to develop social support network, which instill meaning and purpose in life and this may give higher ability to fight negative life challenges. It has been found by Folkman (2008) religious commitment gives meaning in life, purpose, satisfaction and hope in life.

Positive psychological strengths were found to improved subjective well-being of the adolescents. Likewise, previous research by (Khan and Husain^2010^) revealed significant positive relationship between positive psychological strengths and subjective well-being. Apart from that, current finding recommends that problem solving, parental home involvement, gender, socio-economic status, and religiosity of the participants does contribute and have significant influence on positive psychological strengths and subjective well-being. Earlier studies showed that demographic characteristics of the country like gender (Fujita et al.^1991^), religiosity (Frey and Stutzer^2002^) and socio-economic status were found to be strong predictors of one’s life satisfaction (Bradleya and Corwyna^2004^).

Current findings strongly suggest parental involvement was the strongest predictor of positive psychological strengths and subjective well-being (Flouri & Buchanan^2003^) among Indian and Malaysian participants. Parents considered children’s best supporter as both of them their child’s nearest kith and kin. Aunola et al. (2003) declared that a parent’s belief in his or her child’s academic success deeply affected in child’s success and drive to do extremely well. Epstein (2005) suggested understanding in the interaction of parenting skills, student success and commitment to communication consistently influence in student progress. Present research proved that parental involvement improved student well-being (Epstein^2005^).

One of the limitations of the study was that the participants were selected from two cities of each country, which may not be representative of a country as a whole. This study was limited by its reliance on small sample, therefore future research on larger and more diverse samples are required for determining the generalizability of the findings. In addition the measure used in this study although it appears to be valid and reliable instruments, more research is necessary to verify this.

### Ethics statement

All measurements have been carried out on human being complying with local laws.
